# Predicting the contribution of single trait evolution to rescuing a plant population from demographic impacts of climate change

**DOI:** 10.1093/evlett/qraf019

**Published:** 2025-07-08

**Authors:** Diane R Campbell, John M Powers, Justin Kipness

**Affiliations:** Department of Ecology & Evolutionary Biology, University of California, Irvine, United States; Rocky Mountain Biological Laboratory, Crested Butte, United States; Department of Ecology & Evolutionary Biology, University of California, Irvine, United States; Rocky Mountain Biological Laboratory, Crested Butte, United States; Rocky Mountain Biological Laboratory, Crested Butte, United States

**Keywords:** adaptation, environmental stochasticity, evolutionary rescue, phenotypic plasticity, phenotypic selection, specific leaf area

## Abstract

Evolutionary adaptation can allow a population to persist in the face of a new environmental challenge. With many populations now threatened by environmental change, it is important to understand whether this process of evolutionary rescue is feasible under natural conditions, yet work on this topic has been largely theoretical. We used unique long-term data to parameterize deterministic and stochastic models of the contribution of 1 trait to evolutionary rescue using field estimates for the subalpine plant *Ipomopsis aggregata* and hybrids with its close relative *I. tenuituba*. In the absence of evolution or plasticity, the 2 studied populations are projected to go locally extinct due to earlier snowmelt under climate change, which imposes drought conditions. Phenotypic selection on specific leaf area (SLA) was estimated in 12 years and multiple populations. Those data on selection and its environmental sensitivity to annual snowmelt timing in the spring were combined with previous data on heritability of the trait, phenotypic plasticity of the trait, and the impact of snowmelt timing on mean absolute fitness. Selection favored low values of SLA (thicker leaves). The evolutionary response to selection on that single trait was insufficient to allow evolutionary rescue by itself, but in combination with phenotypic plasticity it promoted evolutionary rescue in 1 of the 2 populations. The number of years until population size would stop declining and begin to rise again was heavily dependent upon stochastic environmental changes in snowmelt timing around the trend line. Our study illustrates how field estimates of quantitative genetic parameters can be used to predict the likelihood of evolutionary rescue. Although a complete set of parameter estimates are generally unavailable, it may also be possible to predict the general likelihood of evolutionary rescue based on published ranges for phenotypic selection and heritability and the extent to which early snowmelt impacts fitness.

## Introduction

Climate change is putting many populations at risk of extinction (Bellard et al., 2012; MacLean & Wilson, 2011). At-risk species could persist in the face of climate change by (1) dispersal to new localities with more favorable environmental conditions, (2) altering phenology to match abiotic conditions that change temporally, or (3) expressing new trait values that are adaptive under the new conditions, through a response to selection, phenotypic plasticity, or both. Whereas examples of the first two mechanisms are relatively well known, the third mechanism of persisting in the face of climate change has been less well documented under natural conditions (Parmesan, 2006). With rapid environmental changes around the world, understanding adaptive responses to the changing climate is urgently important, particularly for species with limited dispersal ability, such as many plants.

Adaptive responses to climate change could result from genetic changes due to evolution in response to the new conditions or to phenotypic plasticity (Chevin et al., 2013). The process by which adaptive evolutionary change occurs sufficiently rapidly to counteract a decline in population size under initially unfavorable conditions has been called evolutionary rescue (Gomulkiewicz & Holt, 1995). Evolutionary rescue has been modeled primarily using classical quantitative genetic approaches (Chevin et al., 2010; Chevin et al., 2013; Gomulkiewicz & Holt, 1995; Kopp & Matuszewski, 2014), although there is increasing interest in incorporating genomics (Bay et al., 2017; Urban et al., 2024). The likelihood of evolutionary rescue depends upon the balance between the speed of evolutionary adaptation and the initial maladaptation of the population (Gomulkiewicz & Holt, 1995). A population declines initially in size due to the environmental challenge, then alleles or traits adaptive in the new environment increase in frequency, leading to higher mean absolute fitness and eventually a rebound in population size, if fitness is increased enough before the population goes extinct (Carlson et al., 2014).

Evolutionary rescue can occur under greater environmental change when (1) genetic variance in a trait is high, (2) selection on the trait has a high sensitivity to the environment, and (3) generation time is short (Chevin et al., 2010). Phenotypic plasticity, if adaptive, can accelerate the change in a favored trait, which in turn increases absolute fitness more rapidly, and thereby further promote population persistence if plasticity is not costly to the organism (Scheiner et al., 2019). Despite that theoretical understanding, and laboratory demonstrations with microbes (Bell, 2017), we currently lack much understanding of how often conditions suffice for multicellular organisms in nature (Gomulkiewicz & Shaw, 2013; Urban et al., 2024), but see Peschel & Shaw (2024).

Here, we use a well-studied plant system to determine how evolution in a single trait influences the likelihood of population persistence under climate change. Admittedly, the analysis of a single trait under selection does not capture the full extent of evolutionary rescue, which depends upon the increase in mean overall fitness in the population (Peschel & Shaw, 2024). We chose this approach nevertheless because it illustrates how theoretical models of evolutionary rescue can be applied to real systems in which information on the impact of a single trait is more typically available. At least three kinds of information are necessary: genetic variance, strength and environmental sensitivity of natural selection, and effect of an environmental challenge on mean fitness. All of these are challenging to measure under natural conditions, although information on one or two of them is sometimes available for a given plant species, including estimates of selection on functional traits (Dudley, 1996), genetic variation (Ahrens et al., 2019), or change in mean fitness in a novel environment (Walter et al., 2023). Here, we leverage 12 years of data on natural selection and plasticity, in combination with previously published data, to provide for the first time all three kinds of information for natural plant populations.

We focus on the subalpine plants scarlet gilia, *Ipomopsis aggregata*, and its close congener *I. tenuituba* (Polemoniaceae), in the Rocky Mountains, Colorado, USA. In the western USA including Colorado, there is already a 10%–20% loss in water contained in the snowpack since the 1980s, with a further loss of 60% projected over the next 30 years (Fyfe et al., 2017), and this reduced snowpack has caused shifts toward earlier melting in the spring (Clow, 2010). In the Colorado Rocky Mountains, *Ipomopsis* populations are threatened by earlier snowmelt, which currently causes lower seedling emergence, lower chance of survival to the next year, and lower seed production, projecting declines in local abundance (Campbell, 2019). Although pollen dispersal could introduce new genes that aid persistence, seed dispersal and migration to more suitable habitat are unlikely to contribute to persistence as seeds rarely disperse >1 m (Campbell et al., 2017). On the other hand, several traits of *I. aggregata* experience ongoing natural selection and show genetic variance (Campbell et al., 2022), thus providing the raw material for evolutionary rescue from these deleterious impacts of climate change. One such trait is specific leaf area (SLA): the ratio of leaf area to dry mass. On a global scale, low SLA (i.e., thick leaves) is often associated with dry conditions as it reduces surface area, thereby reducing water loss from the leaf, at the cost of reduced photosynthesis (Poorter et al., 2009). In some natural populations of *Ipomopsis*, plants with low SLA had higher survival (Campbell et al., 2024), demonstrating selection on the trait. In a short-term experimental study of *I. aggregata*, selection on this trait depended upon snowmelt timing in the spring; plants with low SLA were more likely to survive to flower when snowmelt was artificially accelerated, but not so under later snowmelt (Navarro et al., 2022). In a quantitative genetic study in the field, SLA showed significant narrow sense heritability of 10%, indicating its potential to evolve in response to phenotypic selection (Campbell et al., 2022). Finally, the trait shows phenotypic plasticity; in repeated measures of the same plants over several years, SLA is lower in years of earlier snowmelt (Campbell et al., 2022), which is adaptive (Navarro et al., 2022).

We first use 12 years of field data on SLA and its impact on fitness from years that varied greatly in snowmelt timing to determine overall selection on SLA and its environmental sensitivity under natural conditions. Only one rare study of flowering time measured selection in natural plant populations over a longer period (Ehrlén & Valdés, 2020). We then develop models of evolutionary rescue, incorporating the known genetic variance in that trait, and use them to determine whether evolutionary response to the selection is sufficient to counteract the impact of snowmelt timing on population growth. We address four specific questions:

1. How does selection on SLA depend on snowmelt timing?
2. How do magnitudes of heritability and selection intensity affect the likelihood of evolutionary rescue, and do actual field estimates for SLA fall in the range needed for evolutionary rescue?
3. Is population persistence likely given the overall temporal trend toward earlier snowmelt, and how does environmental stochasticity, in the form of variability around that trend, affect persistence? With climate change likely to increase extremes (IPCC, 2023) as well as average temperature, it is important to consider the impact of that variability.
4. How does phenotypic plasticity affect the likelihood of evolutionary rescue in this natural system?

## Methods

### Study system

The study sites consisted of three “Poverty Gulch” sites in Gunnsion National Forest and one site “Vera Falls” at the Rocky Mountain Biological Laboratory (RMBL), all in Gunnison County, CO, USA. At Poverty Gulch, there is a natural hybrid zone between *I. aggregata* ssp. *aggregata* and *I. tenuituba* ssp. *tenuituba* (Campbell et al., 1997). Focal plants included two sets of plants. One set (data from 2009–2019) consisted of plants in common gardens at three sites: an *I. aggregata* site (hereafter “agg”), an *I. tenuituba* site (hereafter “ten”), and a site at the center of the natural hybrid zone (hereafter “hyb”). These sites correspond to sites L, C, and I, respectively, in Campbell et al. (1997) (map in Aldridge & Campbell, 2009). The second set consisted of plants growing in situ at two of the same Poverty Gulch sites (“agg” and “hyb”), and an *I. aggregata* site at Vera Falls (hereafter “VF”; data from 2017–2023). Natural populations of *Ipomopsis* at these sites are relatively small, with typically 30–70 flowering individuals, along with plants in the vegetative state, in a given year.

At these sites, plants of *Ipomopsis* emerge as seedlings in the spring, and spend 2–12+ years as a rosette of leaves before sending up a flowering stalk during the year of flowering (Campbell et al., 2008). The mean generation time is 5 years in this locality (Campbell & Waser, 2007). The plants bloom during a single season, set seed, and then die. The plants have hermaphroditic flowers and are self-incompatible. The primary pollinators are hummingbirds and hawk moths, with occasional flower visits from butterflies and solitary bees (Campbell et al., 1997; Price et al., 2005). The common gardens were started from seed in 2007 and 2008 (details in Campbell, 2019; Campbell & Powers, 2015). Measurements of SLA in these gardens began when plants were 2 years old, either 2009 or 2010 depending upon the garden, as they are only small seedlings during their first summer after seed maturation. By 2018, all but 15 of the 4512 plants originally planted had died, with or without blooming, and we stopped following these gardens. Starting in 2017, in situ vegetative plants at the *I. aggregata* site and the hybrid site whose longest leaf exceeded 25 mm were marked with metal tags to facilitate identification.

### Measurements of trait and fitness

In each year of the study, one leaf from each vegetative plant was collected in the field and transported on ice to the nearby RMBL, 8 km distant. There, each leaf was scanned with a flatbed scanner and analyzed using ImageJ (National Institutes of Health, Bethesda, MD, USA) to measure leaf area. The leaf was dried at 70 °C for 2 hr and then weighed to obtain dry mass and calculate SLA as area/dry mass. For plants in the common gardens, SLA was measured on 982 leaves from 383 plants in 2009–2014. For in situ plants, SLA was measured on one leaf from each of 877 plants in 2017–2022.

Fitness was estimated as the binary variable of survival to flowering. Plants that were still alive in 2019 in the common gardens or in 2023 at the end of the study were assumed to survive to flowering. Although, it is theoretically possible that SLA could also influence flower number or seeds per survivor through effects on resource acquisition during earlier parts of the lifecycle, a previous study of *I. aggregata* found no evidence that selection on SLA differed whether flower number was included or not in the fitness estimate (Navarro et al., 2022).

### Question 1: selection on SLA and its environmental sensitivity

All data analysis and modeling was done in R v.4.4.2. To estimate selection on the trait itself, rather than selection on its yearly plastic responses, we determined the overall average standardized selection differential on SLA. To do so, we first averaged SLA across repeated measurements in multiple years for a given plant. This simplification ignored the extent to which an individual plant matched SLA in a given year to local conditions, but that aspect is partly captured by the addition of plasticity (see below). We then performed generalized linear models of relative fitness (fitness divided by global mean fitness) as a function of mean SLA and the factor of site, after expressing SLA in units of standard deviation by subtracting the mean and dividing by the standard deviation across plants. The within-site regression coefficient for the effect of the standardized trait value on relative fitness (fitness divided by mean fitness) gives the SD-standardized selection differential (Kingsolver et al., 2001), chosen for easy comparison with selection estimates in large reviews of plant functional traits (Caruso et al., 2020; Geber & Griffen, 2003). Site was included as a fixed factor in this model because survival differed on average across the three sites (*p* < 0.0001), ensuring that site-related variation was accounted for in the analysis.

We evaluated the environmental sensitivity of selection on SLA only for the agg and hybrid sites as we had much longer time series for those sites. Our first step was to use individual measurements in each year and find the separate standardized selection differential in each year, including the factor of site along with the effect of standardized SLA in the model. The selection differential was standardized within year but not within site for this analysis. In a second step, we then regressed the selection differential (both standardized and unstandardized in original units of SLA for use in models) on the date of snowmelt in that year. A steeper slope would indicate greater environmental sensitivity of selection. We also examined trends in snowmelt date from 1985–2023 at sites agg, hyb, VF, and the RMBL (Supplementary Methods S1). The RMBL data were included because previous studies of how demography depends on snowmelt timing relied on those values (Campbell, 2019), and we therefore calibrated the evolutionary rescue models in the next sections the same way. Snowmelt date averaged 6 days later at the agg site than at RMBL, 17 days later at the hyb site than at RMBL, and 3 days earlier at site VF, with a common slope of 0.20 days earlier per year.

Most models of evolutionary rescue assume that selection on the trait is stabilizing, with an optimum that moves with the environment (Gomulkiewicz & Houle, 2009). In separate analyses, we also tested for stabilizing selection in each year using a model with site, the standardized trait value and the squared value of the standardized trait value. A negative slope of survival on the squared value would indicate curvature to the fitness relationship that corresponds with stabilizing selection. The quadratic regression coefficients were multiplied by 2 to obtain the quadratic selection gradients (Stinchcombe et al., 2008). Since the fitness component was binary, for all tests we employed function *glm* to perform the generalized linear models with a binomial distribution to test for statistical significance while reporting quantitative estimates of the selection differentials based on ordinary least squares regression (Kingsolver et al., 2012).

### Question 2: modeling dependence of evolutionary rescue on magnitudes of heritability and selection

**Figure 1.**
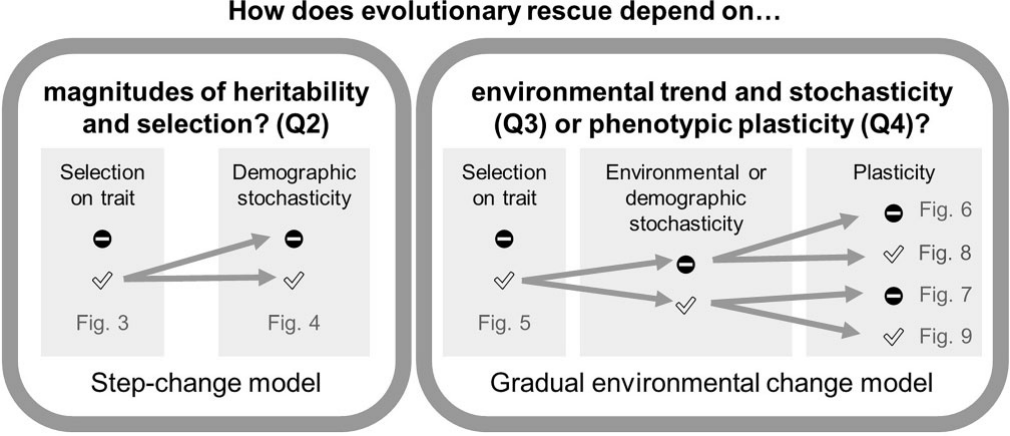
Conceptual diagram showing the elements of the two models and their relationships to the questions addressed and the resulting figures. Left panel: The *Step change model* specifies an abrupt shift to an extreme environment of early snowmelt which is then held constant. Right panel: The *Gradual environmental change model* specifies a linear trend toward earlier snowmelt. The minus sign indicates absence, and the checkmark indicates presence of the feature (e.g., “Plasticity”).

As the observed selection on SLA was always directional, with no significant stabilizing or disruptive selection in any year (see *Results* section), we used iterative models of evolutionary rescue based on directional selection rather than previous models that assumed stabilizing selection. We developed several models of evolutionary rescue, building from simple to complex (Figure 1). Our simplest model (“*Step change model*”) resembled many previous models (e.g., Gomulkiewicz & Holt, 1995; Xu et al., 2023) in describing an abrupt change to a new environment. We used it to examine how much evolutionary response (and hence selection intensity and heritability) was required to counter a particular drop in mean absolute fitness due to early snowmelt, based on a model for directional selection proposed by Campbell (2008):


(1) \begin{eqnarray*}
{{N}_t} = {{\overline{W}}_{t - 1}}{{N}_{t - 1}},
\end{eqnarray*}

(2) \begin{eqnarray*}
{{\overline{W}}_t} = {{\overline{W}}_0}\left[ {1 + \frac{b}{v}\Delta {{z}_t}} \right],
\end{eqnarray*}

where *N*_*t*_ = population size in generation *t*, ${{\overline{W}}_t}$ = mean absolute fitness in generation *t*, and ${{\overline{W}}_0}$ = mean absolute fitness after the environmental challenge but prior to allowing for evolution. The portion in brackets expresses how absolute fitness is altered by the evolutionary response given an abrupt environmental shift to earlier snowmelt. The value $\Delta {{z}_t}$ is the cumulative evolutionary response after *t* generations in the mean value for trait *z*. Following standard quantitative genetic theory 


(3) \begin{eqnarray*}
\Delta {{z}_t} = {{h}^2}S + \Delta {{z}_{t - 1}},
\end{eqnarray*}

where ${{h}^2}$ is heritability and *S* is the selection differential, which in turn is the covariance between relative fitness and the trait value (Falconer & MacKay, 1996). Heritability of SLA was set to 0.10, as estimated in the field at these sites (Campbell et al., 2022). The expression *b/v* converts Δ*z_t_* into an effect on fitness; it equals *b*, the slope of fitness on *z*, divided by mean fitness, *v*. We assumed a starting stable population of 200 individuals based on historical demographic studies of *I. aggregata* (Price et al., 2008; Waser et al., 2010) and then a step change to constant prolonged drought, as in 2012, corresponding to the lowest annual value for absolute fitness observed across 15 years of study by Campbell (2019). That year had the earliest snowmelt date (RMBL day in year = 114) in the study, and we used the average of fitness across the *I. aggregata* site (${{\overline{W}}_0}$ = 0.82) and the hybrid site (${{\overline{W}}_0}$ = 0.93; average = 0.88). These values for fitness were estimated by the intrinsic rate of increase (λ) in integral population models that took into account all stages of the lifecycle from seed to seedling to vegetative plant to flowering plant to seed in the next generation (Campbell, 2019). For the strength of selection on SLA, we first used values from 2010, the year in which we observed the strongest selection favoring low SLA (Table 1). Since selection was estimated from survival, we are assuming that overall fitness would be reduced by the same amount as survival. This *Step change model* corresponds to a situation involving prolonged extreme drought and prolonged strong selection.

**Table 1.** Standardized selection differentials on SLA in each year. Directional selection differentials were estimated from models with a linear term of standardized SLA along with the factor of site. Quadratic selection gradients were estimated by doubling the quadratic regression coefficient in models with linear and quadratic terms of standardized SLA along with site. Statistical departure from zero was assessed using models with a binomial distribution of residuals and was not based on standard errors from the estimates in ordinary least squares regression.

Year	Directional selection differential	Quadratic selection gradient
2009	−0.171 ± 0.081*	0.092 ± 0.108
2010	−0.230 ± 0.087*	−0.004 ± 0.090
2011	−0.039 ± 0.065	0.094 ± 0.050
2012	−0.033 ± 0.067	0.084 ± 0.048
2013	−0.174 ± 0.063**	−0.002 ± 0.074
2014	−0.063 ± 0.060	0.098 ± 0.070
2017	−0.213 ± 0.081*	−0.160 ± 0.122
2018	0.022 ± 0.112	−0.102 ± 0.105
2019	−0.058 ± 0.133	0.268 ± 0.282
2020	0.060 ± 0.135	0.057 ± 0.160
2021	0.125 ± 0.117	0.002 ± 0.135
2022	−0.114 ± 0.075	−0.037 ± 0.109

*p < 0.05; **p < 0.01.

To fully answer question 2, we examined the sensitivity of the results to changes in heritability and the selection intensity, in both cases by stepping the parameter across the entire range of theoretical values, from 0.05 to 1.00 by a step of 0.05. Since the plants are hermaphroditic and self-incompatible, in the deterministic case, we assumed that the population would be functionally extinct if population size fell below 2. To investigate the impact of demographic stochasticity, instead of obtaining the population size in the next generation by multiplying by average fitness, for each individual, we drew the number of individuals in the next generation by sampling from a Poisson distribution with a mean equal to average fitness (λ), as justified in Ellner et al. (2016). The *Step change model* explores the theoretical bounds on evolutionary rescue and thus provides a baseline for understanding how population persistence is affected by a particular drop in mean absolute fitness, but has the disadvantage that the step change in environment is unrealistic.

### Question 3: population persistence given mean and variance of temporal trend in snowmelt

To determine whether evolutionary rescue can occur given empirically grounded estimates of parameters including the projected temporal trends in snowmelt date, we developed the *Gradual environmental change model* (Figure 1) in which snowmelt date was assumed to continue advancing linearly following the trend estimated since 1984 of 0.20 days per year. Because the environmental change is expressed in terms of years rather than generations, we modeled population size over years for this scenario. For simplicity, we retained discrete generations and assumed that the evolutionary response in SLA per year was one fifth as high as the evolutionary response per generation (=5 years). We then modeled the effect separately for the hyb and agg populations, for which we have the longest time series of data. These two populations differ in demography, in that the hybrid population is already below replacement (λ < 1), whereas the *I. aggregata* population currently has λ > 1 but is predicted to fall below replacement in the near future with earlier snowmelt (Campbell, 2019). For these two population, we allowed absolute fitness in the absence of evolution (${{\overline{W}}_0})$ to decline with snowmelt day at RMBL as determined from fitting quadratic models (Table 2) to the results of integrated projection models in a long-term demographic study (Campbell, 2019).

**Table 2.** Parameter expressions used in modeling the impact of a linear trend in snowmelt timing and selection on specific leaf area (*Gradual environmental change model*).

Site	Process	Parameter expression
Both sites	Day of snowmelt (d)	d = 539.2–0.20*t
	SD in predicted snowmelt	11.4d
Hybrid	Absolute fitness in absence of evolution (${{\overline{W}}_0})$	${{\overline{W}}_0}$ = 1.352–0.0202*d + 0.000126*d^2^
	Selection differential (*S*)	*S* = −10.041 + $0.040{{d}_t}$
	Change in trait value by time *t* ($\Delta {{z}_t})$	$\Delta {{z}_t} = \frac{{{{h}^2}( { - 10.041 + 0.040{{d}_t}} )}}{5} + \ \Delta {{z}_{t - 1}}$
	Effect of trait value on absolute fitness relative to the mean ($\frac{{{{b}_t}}}{{{{v}_t}}}$)	$\frac{{{{b}_t}}}{{{{v}_t}}} = ( { - 0.0224 + 0.00012{{d}_t}} )/{{\overline{W}}_0}_t$
*Ipomopsis aggregata*	Absolute fitness in absence of evolution	${{\overline{W}}_0}$ = 7.504– 0.1378*d + 0.00067*d^2^
	Selection differential	*S* = 31.493–0.252${{d}_t}$
	Change in trait value	$\Delta {{z}_t} = \frac{{{{h}^2}( {31.493 - 0.252{{d}_t}} )}}{5} + \ \Delta {{z}_{t - 1}}$
	Increase in absolute fitness relative to the mean	$\frac{{{{b}_t}}}{{{{v}_t}}} = ( {0.0144\ - \ 0.00012{{d}_t}} )/{{\overline{W}}_0}_t$

*Note. d* = day; *t* = year; *h^2^* = heritability; *b* = slope of fitness on trait value *z; v* = mean fitness.

To incorporate the effect of evolution of SLA, we first modeled how the strength of selection on SLA changes with snowmelt day by regressing the selection differential (*S*) on annual snowmelt date for our 12 years of study (Table 2) and substituting that for *S* in Equation 3. As Δ*z* is the change in mean phenotype between two generations and generation time is 5 years, we divided ${{h}^2}S$ by 5 in incrementing it each generation (see expression for $\Delta {{z}_t}$in Table 2). Similarly, to obtain how *b*/*v* (the change in fitness with the trait) changes with snowmelt day, we used the regression coefficient *b* in each year, and regressed that divided by mean survival against snowmelt day (Table 2).

We then used our *Gradual environmental change model* to evaluate the influences of environmental stochasticity and phenotypic plasticity. We added environmental stochasticity in the form of observed variation around the trend line for snowmelt day as a function of year. Using again the linear relationship from Wadgymar et al. (2018), we used the *predict* function in base R to find the residual standard deviation of the predicted values during the time span of 1984–2023. The residual SD measures how much actual snowmelt dates deviate from the predicted values. While there is a general linear trend toward earlier snowmelt, the actual dates vary around that trend with an SD of 11.4 days. So for each year, we drew a value for snowmelt day from a normal distribution with mean as predicted but now with an SD of 11.4 days. Due to the addition of this random element, we ran this model 10 times. To incorporate demographic stochasticity, as in our *Step change model*, for each individual we drew the number of individuals in the next generation by sampling from a Poisson distribution with a mean equal to average fitness (λ).

### Question 4: impact of phenotypic plasticity on population persistence

To investigate the impact of phenotypic plasticity on population persistence, we added plasticity in SLA to the *Gradual environmental change model* both with and without stochastic environmental variability around the temporal trend line in snowmelt. To do so, we used an equation for SLA as a function of snowmelt day for a set of plants that were measured repeatedly in different years by Campbell et al. (2022). Thus, plasticity was modeled as a linear function of the present environment as detected by the individual plant (Greenspoon & Spencer, 2021). Using repeated measures ANOVA, SLA increased by 1.323 cm^2^/g for every day later the snow melted. Assuming no cost of reduced SLA, to our prediction for the trait value in a given year, we added 1.323 times the difference in snowmelt day from the previous year.

## Results

### Question 1: selection on SLA

**Figure 2.**
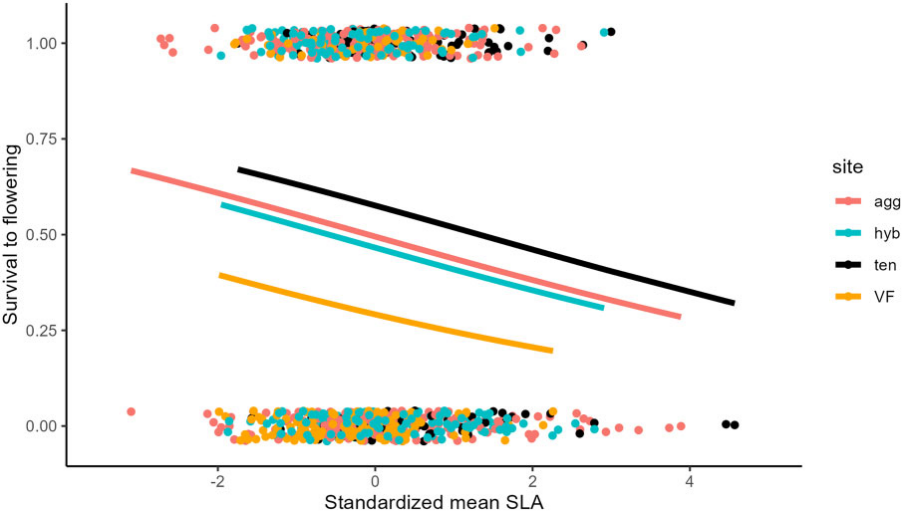
Survival to flowering as a function of mean specific leaf area (SLA) for a plant. Points are jiggered for visibility; all values for survival = 0 or 1. Lines are predicted values obtained by inverse prediction from a generalized linear model with a binomial distribution: glm(survtoflr ∼ site + rsla, family = binomial). These are bounded by 1 and 0 but appear approximately linear over the range of observed SLA. Sites “agg” and “VF” both contain *Ipomopsis aggregata*. Site “ten” contains *I. tenuituba*, and site “hyb” contains natural hybrids between the two parental species.

**Figure 3.**
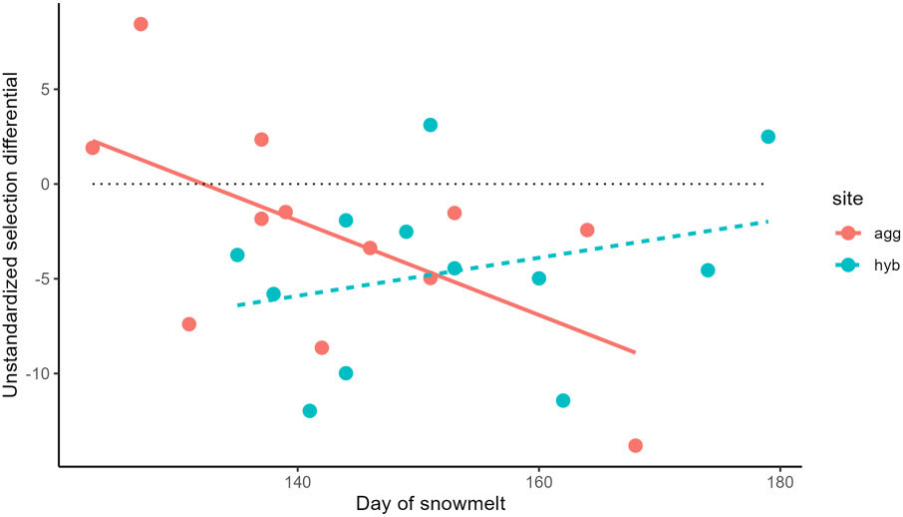
Unstandardized selection differential as a function of day of snowmelt in two sites. The models were originally fit to Rocky Mountain Biological Laboratory day of snowmelt and then adjusted by a constant to reflect accurate estimates of snowmelt in the individual sites. The selection estimate was negative (below the dotted line) in 19 of 24 site-year combinations. Selection decreased with day of snowmelt in the *agg* site (*p* < 0.05; solid line), but did not change significantly with day of snowmelt in the hybrid site (dashed line).

Using mean values for SLA for individual plants, the overall standardized selection differential was *S*' = −0.12 (SE = 0.04, *p* = 0.0012; Figure 2) for effect of the trait in a model that also included site, as compared with the values of *S*' = −0.33 and −0.19 (*p* = 0.007) in *I. aggregata* and *I. tenuituba* sites measured previously in 2009–2014 only (Campbell et al., 2024). These values all indicate that lower values (thicker leaves) led to higher survival to flowering. Selection was detectably different from zero in 4 years (Table 1), with the strongest value of *S*' = −0.23 (SE = 0.08, *p* < 0.01) in 2010. The selection differential (*S*) did not correlate significantly with snowmelt date in the spring (*r* = −0.20, *p* > 0.05), but was negative, favoring thicker leaves, in 19 of 24 site-year combinations (Figure 3). When broken up by site, it showed a positive trend (but not significant) at the hybrid site only; in other words in the direction expected with stronger selection for lower SLA when snowmelt was earlier (Figure 3).

### Question 2: modeling dependence of evolutionary rescue on magnitudes of heritability and selection

**Figure 4.**
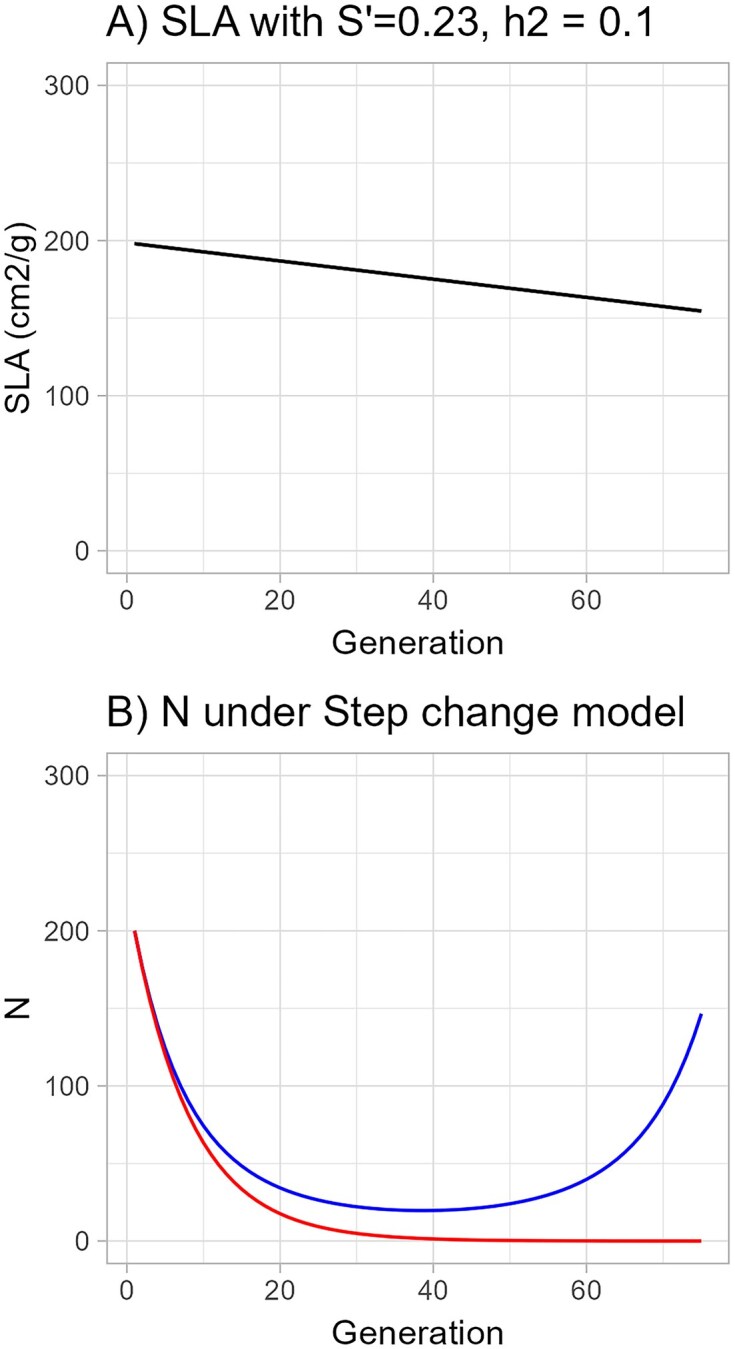
*Step change model* with shift to a constant extreme environment. (A) Evolution of specific leaf area (SLA) with constant selection (S' = 0.23) and heritability = 0.10. (B) Change in population size (*N*) for the case of no selection on SLA (red line) and case of constant selection on SLA (blue line). In 2010, the year with strongest selection for smaller SLA, mean survival (*v*) = 0.554, and the regression coefficient (*b*) of absolute survival on raw SLA was −0.0034, making b/v (the relative amount by which absolute fitness is changed per cm^2^/g increase in SLA) = −0.0061. The selection differential (S) obtained from the covariance of relative survival with raw SLA was −5.866, and heritability was assumed to be 0.10, making Δ*z* = −0.59, a drop in SLA of 0.59 cm^2^/g per generation.

With the assumption that early snowmelt made ${{\overline{W}}_0}$ = 0.88, in the absence of the evolution shown in Figure 4A, a population starting at size 200 would fall below 2 (the minimum that can perpetuate a self-incompatible species) and thus be functionally extinct by the 38th generation (Figure 4B). With the predicted evolution of SLA toward lower values in response to the strongest selection observed (*S*' = −0.23 and *Step change model*; Figure 4A), the expected population size fell only as low as 20 individuals (in generations 34–44) before rising again as mean fitness crossed zero and eventually followed exponential growth to above its starting level, showing evolutionary rescue (Figure 4B). With demographic stochasticity added, 4 of 10 populations starting at size 200 went extinct by the year 2400 (75 generations; Supplementary Figure S1).

**Figure 5.**
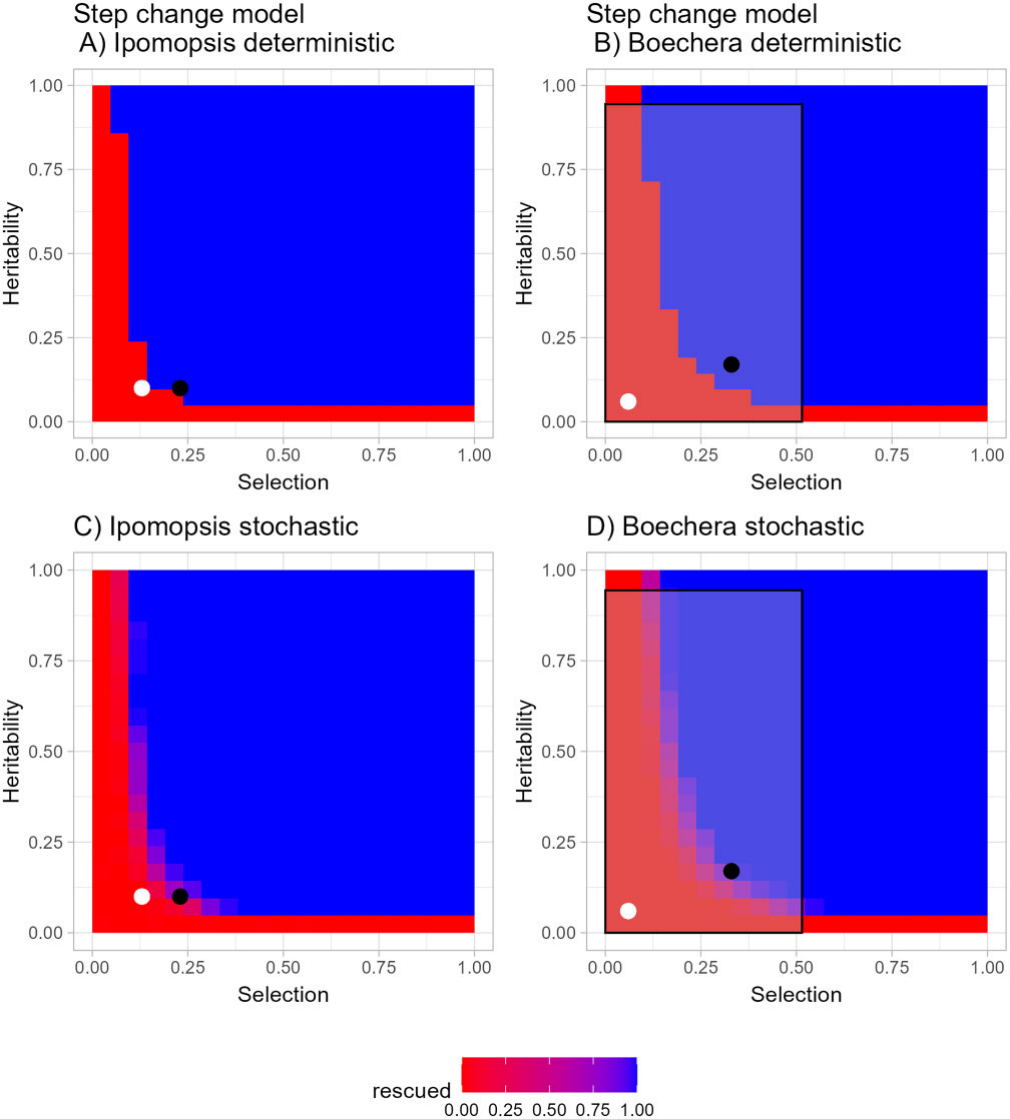
Parameter space for heritability and standardized selection differential showing conditions allowing evolutionary rescue in response to a constant extreme environment (*Step change model*). Starting population size = 200. Red indicates extinction. Blue indicates evolutionary rescue by generation 75. Deterministic models shown for (A) mean absolute fitness under early snowmelt in the absence of evolution = 0.88 in *Ipomopsis aggregata* and (B) 0.79 in *Boechera stricta*. Points for species are plotted at their field-estimated parameter values. For *Ipomopsis* (A and C), the black circle indicates the strongest selection observed (S' = 0.23) and the white circle indicates mean selection (S' = 0.13). For *Boechera* (B and D), the two points correspond to two sets of parameters from different common garden experiments (see Supplementary Methods S2). Panels (C) and (D) include demographic stochasticity. In that case, the shading indicates the proportion of runs in which the population was rescued. Approximately 90% of runs for *Ipomopsis* values went extinct when S' = 0.13. The rectangle in (B) and (D) encompasses a 95% confidence interval around the mean values for the selection differential and heritability in a review of plant functional traits (Geber & Griffen, 2003).

Increasing the heritability would increase the likelihood of evolutionary rescue (Figure 5A), whereas halving it would prevent it, given that a population starting at 200 individuals would drop below 2 by generation 66 and thus be functionally extinct at that time (Supplementary Figure S2). Doubling the selection intensity in the same way has an even larger effect (Figure 5 and Supplementary Figure S2) because it not only doubles the rate at which the trait value changes but also increases the influence of that change on absolute fitness.

Using the overall average value for selection (*S*' = −0.13), as the best estimate, rather than its value of −0.23 in the most extreme year of 2010, the estimated heritability would put *Ipomopsis* right on the dividing line between local extinction and population persistence in a deterministic model (Figure 5A). With demographic stochasticity added, only 10% of simulated *Ipomopsis* populations persisted to generation 80 (white circle in Figure 5C). All of these models so far examined evolutionary rescue only in the face of an abrupt shift to earlier snowmelt.

### Question 3: population persistence given mean and variance of temporal trend in snowmelt

**Figure 6.**
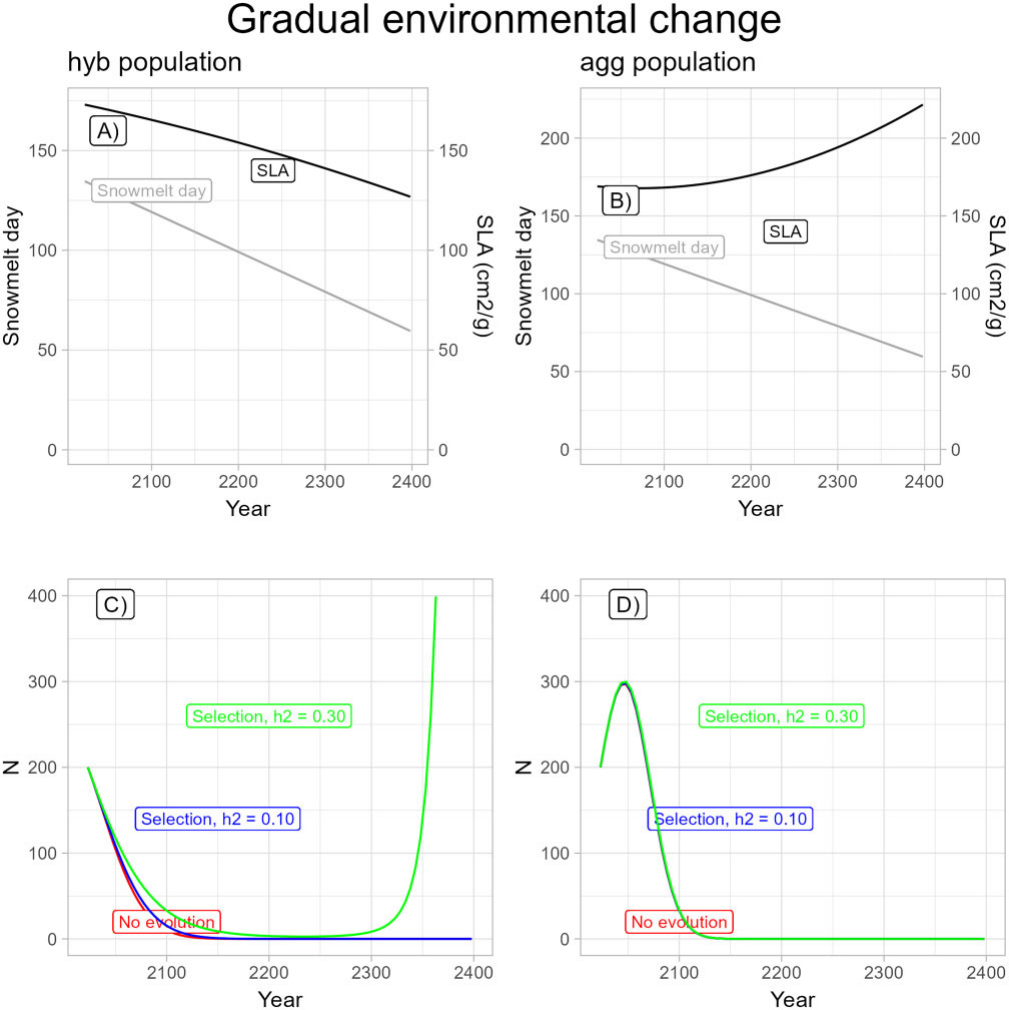
Predictions from the *Gradual environmental change model* when selection on specific leaf area (SLA) is included but not plasticity or stochasticity. Snowmelt date (days since January 1) and the evolutionary response of SLA depicted with light and dark lines, respectively, for the (A) hybrid population and (B) *Ipomopsis aggregata* population. Population size (*N*) for the case of no trait evolution (red line), selection with observed heritability of 0.10 (blue line), and selection with heritability of 0.30 (green line) for approximately 75 generations shown for the hybrid population (C) and *I. aggregata* population (D).

The *Gradual environmental change model* allowed a more realistic continuous change in the day of snowmelt in the spring (Figure 6A and B) rather than a shift to extreme drought. In this case, in the absence of evolution, a hybrid population starting at size 200 would fall below 2 in the year 2128 and thus be functionally extinct by the 21st generation (Figure 6C). Allowing for the observed trend in selection on SLA would not allow evolutionary rescue at the hybrid site, as the population size would still fall below 2, in this case in 2138 and stay below 2 for a very lengthy time (blue line in Figure 6C shows population size still decreasing in the year 2400). Thus adding the extra realism of the actual trendline for snowmelt timing and selection eliminated the opportunity for evolutionary rescue. Even though the initial environmental hit to average survival in generation 1 was less strong with gradual environmental change than in the *Step change model* (mean fitness in generation 1 = 0.92 as compared with 0.88), selection on SLA was weaker at that point and would not reach the value used in the *Step change model* until the year 2175 (Supplementary Table S1). Increasing heritability to 0.30 would make evolutionary rescue possible even in this more realistic scenario, as a hybrid population starting at size 200 would not drop below 3 individuals (green line in Figure 6C).

In contrast, the *Gradual environmental change model* with a realistic trendline for snowmelt in an *I. aggregata* population showed very little effect of heritability on population persistence (Figure 6B). This population is not yet below replacement (Campbell, 2019), so allowing for selection and using the best fitting quadratic relationship for how mean fitness has previously changed with snowmelt, the population would initially increase in size, but start to decline by 2045 as fitness drops below 1 due to earlier snowmelt. Selection on SLA is not only weaker at the start in this population than in the *I. aggregata* population, but it also trends toward favoring larger SLA with earlier snowmelt in this population (Figure 3). Even raising heritability of SLA to 0.30 would have little effect on the population dynamics, and the population is predicted to drop below its starting value by 2073 and be functionally extinct (*N* < 2) by around 2128, a value similar to that for the hybrid population (Figure 6D) despite the difference in population dynamics to arrive at that point.

**Figure 7.**
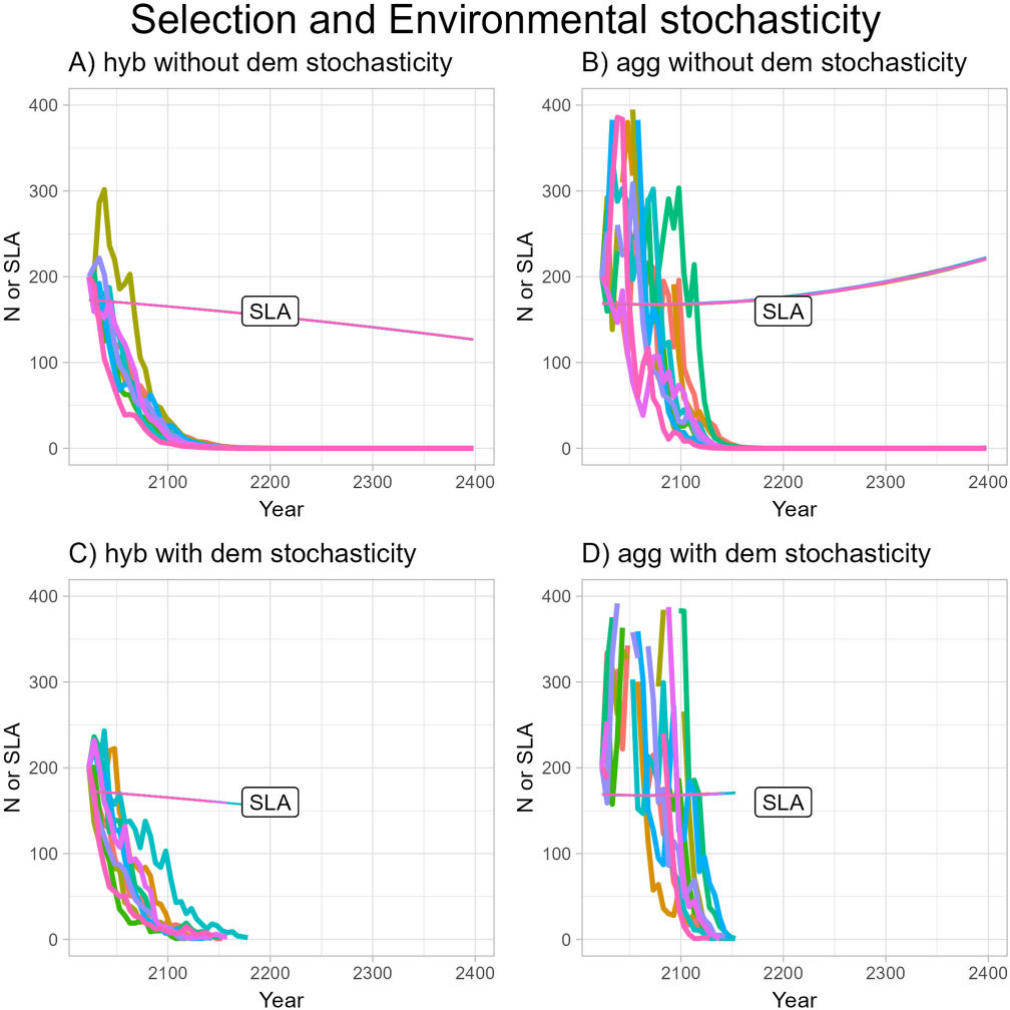
Predicted population size and the evolutionary response of specific leaf area (SLA) as a function of year with environmental stochasticity in the form of variance in snowmelt around the trendline. The model includes selection on SLA but not phenotypic plasticity. Heritability was set at 0.10. The response of SLA is shown in the same color as the population size for the corresponding run but with thinner lines. A separate line is plotted for each of 10 replicates for (A) hybrid population without demographic stochasticity, (B) *Ipomopsis aggregata* population without demographic stochasticity, (C) hybrid population with demographic stochasticity, and (D) *I. aggregata* population with demographic stochasticity. Notice that SLA varied relatively little among replicates, making those lines mostly overlapping.

Adding variability in prediction of snowmelt date did not change the basic pattern that both populations would go extinct in the absence of plasticity (Figure 7). Variability in snowmelt date had, however, more effect on the dynamics in the *I. aggregata* population, causing extreme swings in population size in the first several decades that would greatly change the predicted date for population extinction (Figure 7B). The two populations showed different magnitudes of response to variability in snowmelt date because fitness in the absence of evolution is more sensitive to snowmelt date in the *I. aggregata* population. Allowing for demographic stochasticity had little effect except that all very small populations were predicted to go locally extinct by 2180 (Figure 7C and D).

### Question 4: impact of phenotypic plasticity on population persistence

**Figure 8.**
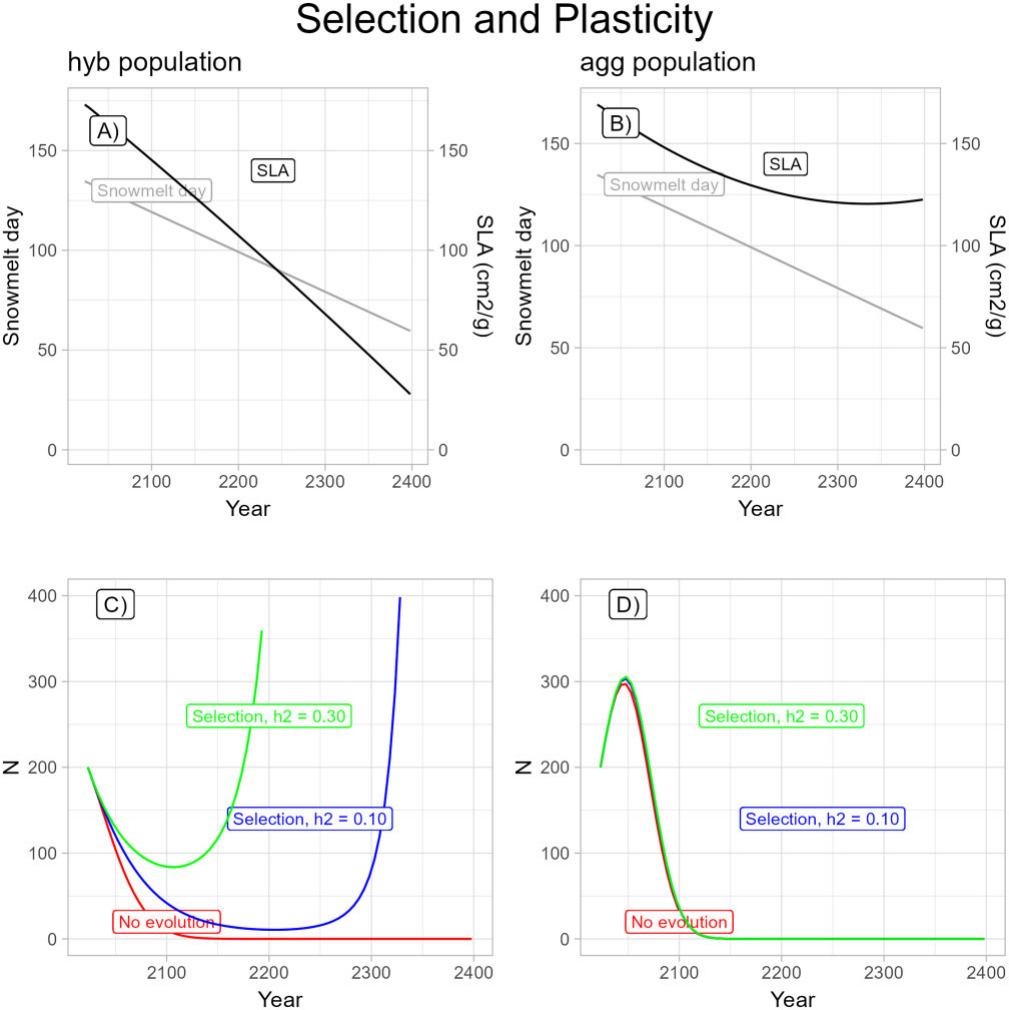
Predictions for selection with added plasticity but no stochasticity in the *Gradual environmental change model*. Starting population size = 200. Snowmelt date (days since January 1) and the evolutionary response of specific leaf area shown for the (A) hybrid population and (B) *Ipomopsis aggregata* population. Population size (*N*) for the case of no selection (red line), selection with observed heritability of 0.10 (blue line), and selection with heritability of 0.30 (green line) for approximately 75 generations shown for the hybrid population (C) and *I. aggregata* population (D).

**Figure 9.**
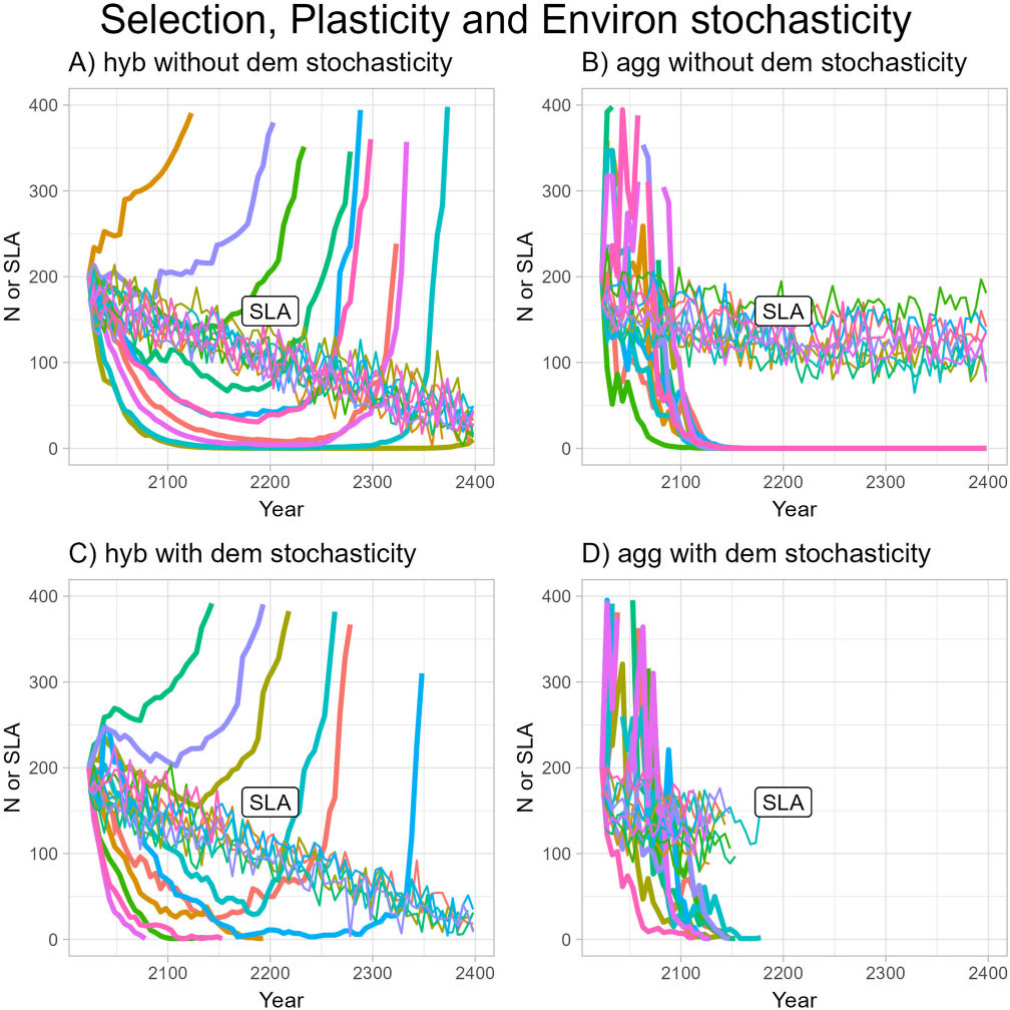
Predicted size of population (*N*) and mean specific leaf area (SLA) (cm^2^/g) as a function of generation with selection, phenotypic plasticity, and environmental stochasticity in the form of variance in snowmelt around the trendline. The observed heritability of 0.10 was used. All populations started with size *N* = 200. The response of SLA is shown in the same color as the population size for the corresponding run but with thinner lines. Ten sample runs are shown for (A) the hybrid population and (B) the *Ipomopsis aggregata* population without demographic stochasticity and (C) the hybrid population and (D) the *I. aggregata* population with demographic stochasticity.

Adding phenotypic plasticity of SLA had a large effect for the hybrid population, allowing the simulated population to persist, never dropping below *N* = 10, when it had not in its absence, and allowing the population to grow starting in 2202 (compare blue line in Figure 8C with blue line in Figure 6C). This was not so for the *I. aggregata* population; even though phenotypic plasticity now made SLA decrease over time, it did not do so rapidly enough to generate evolutionary rescue (Figure 8). For the hybrid population, adding environmental stochasticity in snowmelt timing on top of plasticity, the number of years until population growth became positive was extremely variable across runs of the model, spanning a range from the start until approximately 2300 (Figure 9A). The variation arose because strong phenotypic plasticity made SLA highly responsive to the yearly fluctuations in snowmelt. Number of years until population growth became positive was highly responsive to initial changes in SLA, with a few early low values leading to early evolutionary rescue due to strong increases in absolute fitness (see brown line in Figure 9A). Even with demographic stochasticity, however, the population had a 60% chance of being rescued by evolution in this case (Figure 9C), in comparison with the case without plasticity in which the hybrid population always went extinct (Figure 7C). Adding environmental and/or demographic stochasticity on top of selection and plasticity still did not allow evolutionary rescue in the *I. aggregata* population (Figure 9B and D).

## Discussion

Climate change is projected to cause earlier snowmelt in mountains around the world due to higher temperatures and reduced snowpacks (Clow, 2010; IPCC, 2023; Kraaijenbrink et al., 2021). Here, we modeled how earlier snowmelt affects absolute fitness, trait evolution, and the impact of evolution of a single trait on population persistence in a subalpine herb using field-based parameter estimates. A key finding was that adaptive phenotypic plasticity, in combination with an evolutionary response to selection, was necessary to achieve evolutionary rescue if only that one trait is considered. Our simplest model (*Basic model*) confirmed results of other models in which evolutionary rescue is more likely with higher heritability and lower initial maladaptation (Gomulkiewicz & Holt, 1995). Using field estimates of parameters, and a gradual trend in snowmelt date (*Gradual environmental change model*), we projected local population extinction in the absence of phenotypic plasticity even if adaptive evolution of the trait of SLA is allowed. Adding adaptive phenotypic plasticity would, however, likely rescue one of the two populations modeled because the adaptive phenotypic plasticity increases the rate at which the trait approaches its optimum. The simulated hybrid population is currently below replacement but is projected to be rescued by evolution of that trait due to the combination of strong selection and plasticity. Environmental stochasticity interacted with the change in phenotype, which allowed for very wide variation in the time required for evolutionary rescue in the hybrid population to take place. Thus, even though we predicted that the hybrid population could eventually be rescued by selection and adaptive plasticity in SLA, it is not currently possible to predict for a given local population precisely when population growth would become positive again. These results show that real populations can fall in the parameter space where levels of stochasticity greatly influence population persistence. Although the simulated *I. aggregata* population is currently growing, it is projected to fall below replacement due to earlier snowmelt, and even in the presence of phenotypic plasticity, the weaker selection on SLA in that population is likely insufficient to rescue the population. Thus, in this case, the population most threatened in the near term is the one with better prospects for long-term persistence. Note that these models predict local population extinctions, not species extinction. *I. aggregata* is widespread and common across the mountains of the western USA, encompassing a very wide range of environmental conditions (Grant & Wilken, 1986). Furthermore, there is evidence that recruitment of vegetative rosettes is enhanced by soil disturbance, suggesting that populations may have historically arisen and went locally extinct (Juenger & Bergelson, 2000).

Our models pertain to the effects of only a single trait and are relatively simplistic in comparison with some models of evolutionary rescue (Xu et al., 2023), but we viewed that as necessary to fit to the kind of information typically available. In developing the models, we made many key assumptions. First, we assumed that climate change can cause snowmelt to get progressively earlier following its current linear fit trend over the past four decades, and that no other environmental changes will affect these populations. While recognizing that simplicity, and the increase in extinction that can be caused by a nonlinear environmental change (Greenspoon & Spencer, 2021), we view incorporation of alternative climate scenarios (Thomas et al., 2004) as beyond the scope of this paper. Second, we assumed natural selection was linear, which may be true in the short-term but is unlikely in the longer term as SLA approaches its optimal value for survival. As SLA gets lower, surface area for photosynthesis decreases, and at some point carbon assimilation may then limit fitness more than reduction of water loss. This could occur if a plant escapes drought by rapid growth early in the season. Tests of this drought escape strategy in other species have produced mixed results (Sherrard & Maherali, 2006; Wolfe & Tonsor, 2014). Third, we assumed that phenotypic plasticity would continue indefinitely along a linear trend, and that heritability of the vegetative trait would not change with earlier snowmelt, even though changes with environment are common (Etterson, 2004; Sherrard et al., 2009). Although unlikely in the very long term, SLA in our models with plasticity in most cases did not fall below the minimum observed value (81 cm^2^/g) in a recent study of *I. aggregata* (Navarro et al., 2022) until after absolute fitness rose above 1 causing population size to increase (Figure 8A). Fourth, we assumed that allowing for variation in time to flowering and thus overlapping generations would not alter population dynamics significantly. Fifth, we assumed no cost to phenotypic plasticity, as defined by a fitness decrement of a highly plastic genotype relative to a less plastic genotype, an assumption that may often be met (Murren et al., 2015). Six, we had to make some assumptions about initial population size and when a small population would go extinct, either if population size dropped below 2, or with number of surviving offspring following a Poisson distribution. The populations we studied are small, with 30–70 flowering individuals, along with vegetative individuals, in a given year, and local extinction was not very sensitive to population size between 100 and 200. But demographic stochasticity would cause local extinction even in some cases when population size could otherwise eventually recover, and neither extinction criterion captures fully the reality of variation in lifetime fitness (Shaw et al., 2008).

In addition to these assumptions for a closed population, we also assumed no gene flow or dispersal between populations. Gene flow via pollen could introduce genes that increase adaptation (Wolf & Campbell, 1995). Seed dispersal on the other hand is very limited with seeds rarely moving >1 m (Campbell et al., 2017). The two populations modeled here are 700 m apart and differ in snowmelt date by 11 days on average, meaning that 64 years of seed dispersal upslope would be required to gain just one day later of snowmelt, which is insufficient to keep up with the trend in snowmelt timing.

The information needed to project population persistence with evolution is rarely available. But for *Ipomopsis*, we know both the agent of selection (early snowmelt) and a target of selection (SLA). We have long-term estimates of how snowmelt timing affects mean absolute fitness (Campbell, 2019), heritabilities of some functional traits (Campbell et al., 2022), and now 12 years of selection estimates, allowing us to estimate environmental sensitivity. We are unaware of other plant systems for which all of these data are available, but a few cases come close. For example, data on genetic variation and selection were combined to compare the number of generations for northern populations of *Primula nutans* to evolve some traits of southern populations separated by a temperature difference that climate change could produce in 70–140 years (Mattila et al., 2024). Besides *Ipomopsis*, we are aware of one other plant system for which similar information on the three parameters in our *Step change model* (drop in mean absolute fitness, selection, and heritability) are available: *Boechera stricta*, another herbaceous species in the Colorado Rocky Mountains. We repeated the deterministic and stochastic versions of that model with two sets of parameter estimates for *B. stricta* (Supplementary Methods S2). Conditions appear to allow for evolutionary rescue in some parts of the range based on the earlier study (black circles in Figure 5C and D; Wadgymar et al., 2017) but not under snow removal conditions that mimic climate change (white circles in Figure 5C and D; Bemmels & Anderson, 2019). Extending the model to other plants, we plotted 95% confidence intervals for 653 selection differentials and 1214 estimates of heritabilities for functional traits reviewed by Geber & Griffen (2003). The majority of the space, but by no means all, overlapped with conditions where evolutionary rescue could take place (Figure 5B), with the important caveat that an abrupt shift to an environment resulting in mean absolute fitness of 0.79 is assumed (as that parameter is generally unknown) and the additional caveat that many of the studies estimated heritability in a greenhouse or growth chamber where values were higher than in the field (mean = 0.42 vs. 0.12). Furthermore, only heritability or selection, not both, is known for vegetative traits in most species.

Our models incorporated adaptive evolution and plasticity of only one quantitative trait. All else equal, adding more traits could increase the potential for evolutionary rescue by increasing absolute fitness more quickly, provided the direction of selection is not antagonistic to genetic correlations and phenotypic plasticity of those traits is neutral or adaptive. Note that we chose the trait of SLA because of prior evidence that it was under strong natural selection in the field environments (Campbell et al., 2024) and was plastic with respect to snowmelt timing in a concordant direction (Campbell et al., 2022). A recent meta-analysis found that plastic traits tend to have more additive genetic variation and thus higher evolvability (Noble et al., 2019). Some other traits of *Ipomopsis* that are also under selection may not show plasticity, or may not show it in an adaptive direction. For example, earlier snowmelt leads to smaller flowers and reduced nectar production and yet those trait values are expected to reduce pollination and seed production (Powers et al., 2022). Furthermore, selection on flower length has gotten weaker over time with earlier snowmelt (Campbell & Powers, 2015). We are currently testing how allowing for multivariate trait evolution on several vegetative and floral traits known to affect fitness in *Ipomopsis* would influence evolutionary rescue.

When the traits that affect fitness are unknown, one way to evaluate the overall potential for evolutionary rescue is to estimate the increase in mean fitness as the upper bound on the rate of adaptation, following Fisher’s fundamental theorem of natural selection (Fisher, 1930):


(4) \begin{eqnarray*}
{{\overline{W}}_{t + 1}} = \ {{\overline{W}}_t} + \frac{{{{V}_\mathrm{ A}}\left( W \right)}}{{{{{\overline{W}}}_t}}},
\end{eqnarray*}

where ${{V}_\mathrm{ A}}( W )$ = additive genetic variance in fitness (Peschel & Shaw, 2024). The additive genetic coefficient of variation for fitness $\frac{{{{V}_\mathrm{ A}}( W )}}{{{{{\overline{W}}}_t}}}$ was previously estimated in a quantitative genetic field study of *I. aggregata* in other populations and years from those studied here as 26% based on survival from seedling to flowering, the fitness component most similar to the one in this study, or 15% based on total fitness from seed to seed (neither significantly different from zero; Campbell, 1997). Adaptation per generation in our models was much lower than those estimates of the theoretical maximum. For example, in generation 1 it equaled 0.4% in the *Step change model* (Supplementary Table S1) Although the studies were done in different places and times, the large difference suggests there are other heritable traits with strong effects on fitness in this system (see also Campbell, 1996). Our intent here was only to model how changes in a single trait could contribute to evolutionary rescue, but based on that high estimate for genetic variance in absolute fitness, total evolution might increase the chances of evolutionary rescue in this system.

We modeled expected evolutionary rescue with a quantitative genetic approach. Another promising approach is to use genomics and perform genomic sequencing over time (Urban et al., 2024). A few studies have detected genome evolution by comparisons over time, as in the European great tit (Stonehouse et al., 2023) or over different habitats that differ in ways expected under climate change, as in corals (Bay et al., 2017). These results have occasionally been used to make projections for persistence under climate change, but notably they have had to assume how many loci are involved in thermal tolerance and affect fitness in the field (Bay et al., 2017), whereas we had field data on how the trait affected fitness in particular years. This is one advantage of the quantitative genetic approach; whereas it does not identify particular loci, it is easier to measure field impacts on fitness, as shown by hundreds of studies (Kingsolver et al., 2012) and also how mean fitness changes over time (Shaw, 2019). That may make it a more feasible way to add evolutionary potential to extinction-risk assessments (Forester et al., 2022).

## Conclusions

Using a long-term study of natural selection in the field, in combination with prior field information on heritability and mean absolute fitness, we were able to show that evolutionary rescue of a plant population due to evolution of one trait (SLA) is possible, if we also allow for the high phenotypic plasticity in the trait. Selection and plasticity in combination were projected to rescue one of two populations, and it was the population currently more threatened in which selection was strongest and evolutionary rescue appeared most likely. Our work provides one of the first examples to estimate the major parameters in evolutionary rescue models under natural conditions.

## Supplementary Material

qraf019_Supplemental_File

## Data Availability

Data and code are available on Dryad: https://doi.org/10.5061/dryad.ht76hdrtn.
