# Supplementary material for: Predicting the contribution of single trait evolution to rescuing a plant population from demographic impacts of climate change
Source: Evol Lett. 2025 Jul 8;9(5):533–47. doi: 10.1093/evlett/qraf019 (PMC12492211; doi:10.1093/evlett/qraf019)
Supplement: qraf019_Supplemental_File [file qraf019_supplemental_file.pdf]

Supplementary Materials

Methods S1. Snowmelt timing

We examined trends in snowmelt date (date of bare ground) from 1985-2023 at sites agg, hyb, and VF and 1984-2022 at the Rocky Mountain Biological Laboratory, 8 km distant and at a similar elevation to our *I. aggregata* site (http://www.gothicwx.org/ground-cover.html). The RMBL data were included because previous studies of how demography depends on snowmelt timing that are incorporated into modeling here relied on those values (D R Campbell, 2019), and we therefore calibrated the evolutionary rescue models in the next sections the same way. Snowmelt timing at the two actual sites (agg and hyb) differs only by a constant, as shown by the following analysis. We obtained estimates of snowmelt date at each site in each year from maps of snow persistence prepared by I. Breckheimer from an analysis of Landsat data and the size of the snowpack measured in snow water equivalent near snow telemetry sites (https://arcg.is/1yzKDG). In a model of snowmelt day as a function of year, site (RMBL, agg, hyb, VF) and the site x year interaction, the interaction was not significant ($F_{3, 144} = 0.06$, $P = 0.9800$). Removing the interaction from the model, snowmelt date was 6 days later at the agg site than at RMBL, 17 days later at the hyb site than at RMBL, and 3 days earlier at site VF, with a common slope of 0.20 days earlier per year (SE = 0.09, P = 0.0294).

Methods S2. Evolutionary rescue in *Boechera stricta*

Information on the three parameters needed for the model of extreme drought and prolonged selection (drop in mean absolute fitness, selection, and heritability) is available for another species in the Colorado Rocky Mountains, *Boechera stricta* (Brassicaceae). This small mustard is primarily self-pollinating, but for comparison with *Ipomopsis*, we still considered

population extinction to occur if N < 2. At an elevation of 3133 m, the species has historically stable population size, but in more recent common garden studies, $\lambda = 0.79$ (Anderson & Wadgymar, 2020). One set of parameter values came from (Wadgymar et al., 2017) who estimated significant heritability of SLA as 0.17 (mean of three provided estimates) and significant standardized selection intensity at -0.33. The second set came from snow removal plots mimicking climate change conditions that found little evidence for an evolutionary response in SLA and non-significant heritability of 0.06 (mean of three estimates; (Bemmels & Anderson, 2019)). We repeated the deterministic and stochastic versions of the model for a constant extreme drought and selection (*Basic model*) with these parameter estimates and compared the results with those for *Ipomopsis* using its overall average standardized selection differential on SLA.

References for Supplementary Material

Anderson, J. T., & Wadgymar, S. M. (2020). Climate change disrupts local adaptation and favours upslope migration. *Ecology Letters, 23*, 181-192.

Bemmels, J. B., & Anderson, J. T. (2019). Climate change shifts natural selection and the adaptive potential of the perennial forb *Boechera stricta* in the Rocky Mountains. *Evolution, 73*, 2247-2262.

Campbell, D. R. (2019). Early snowmelt projected to cause population decline in a subalpine plant. *Proceedings of the National Academy of Sciences USA, 116*(26), 12901-12906.

Wadgymar, S. M., Daws, C., & Anderson, J. T. (2017). Integrating viability and fecundity selection to illuminate the adaptive nature of genetic clines. *Evolution Letters, 1*, 26-39.

Table S1. Intermediate dynamics in iterative models for population size. Heritability of the trait was set at 0.10, and starting population size at 200. Adaptation (increase in mean fitness) is given by $\frac{b}{v}\Delta z_t$. $\overline{W}_0$ = mean absolute fitness in absence of evolution.

Step change model: Constant extreme environment and constant selection							
Generation	Year	$\overline{W}_0$	S	$\frac{b}{v}$	Δz_t	$\frac{b}{v}\Delta z_t$	N with evolution
1	2028	0.88	-5.866	-0.0061	-0.587	.0035807	176.6
2	2033	0.88	-5.866	-0.0061	-1.174	.0071614	156.5
3	2038	0.88	-5.866	-0.0061	-1.761	0.0107421	139.2
4	2043	0.88	-5.866	-0.0061	-2.348	0.0143228	124.3
5	2048	0.88	-5.866	-0.0061	-2.935	0.0179305	111.3
6	2053	0.88	-5.866	-0.0061	-3.522	0.0214842	100.1
7	2058	0.88	-5.866	-0.0061	-4.109	0.0250649	90.3
8	2063	0.88	-5.866	-0.0061	-4.696	0.0286456	81.7
9	2068	0.88	-5.866	-0.0061	-5.283	0.0322263	74.2
10	2073	0.88	-5.866	-0.0061	-5.870	0.035807	67.7
Gradual environmental change model: Mean fitness and selection change with snowmelt date as in hybrid population.							
Generation	Year	$\overline{W}_0$	S	$\frac{b}{v}$	Δz_t	$\frac{b}{v}\Delta z_t$	N with evolution

1	2028	0.905	-4.697	-0.0064	-0.56	0.003584	183.3
2	2033	0.889	-4.737	-0.0065	-1.03	0.006695	166
3	2038	0.876	-4.777	-0.0066	-1.51	0.009966	148.5
4	2043	0.863	-4.817	-0.0067	-1.99	0.013333	131.4
5	2048	0.850	-4.857	-0.0068	-2.47	0.016796	114.9
6	2053	0.838	-4.897	-0.0070	-2.96	0.02072	99.3
7	2058	0.826	-4.937	-0.0071	-3.45	0.024495	85
8	2063	0.814	-4.977	-0.0072	-3.95	0.02844	71.9
9	2068	0.802	-5.017	-0.0073	-4.45	0.032485	60.2
10	2073	0.791	-5.057	-0.0074	-4.95	0.03663	49.9

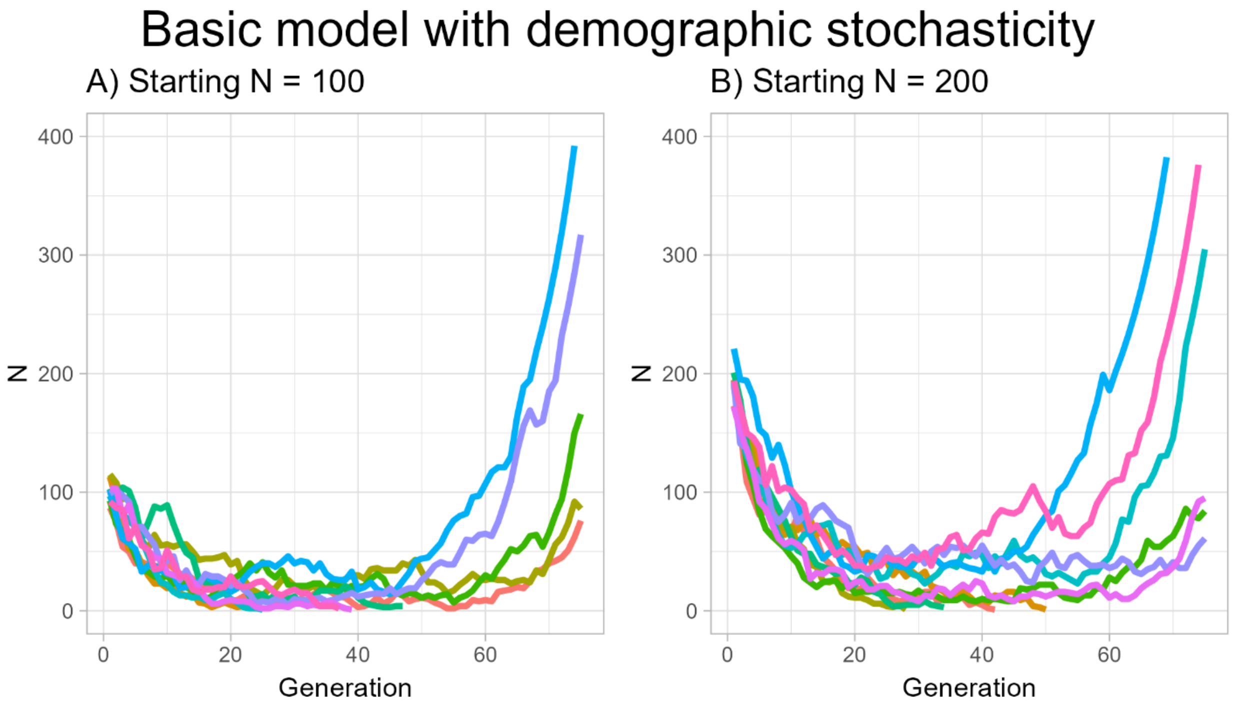

Supplementary Figure S1. *Step change model* of constant selection in an extreme environment with demographic stochasticity added. Ten sample runs are shown starting with (A) N = 100 or (B) N = 200. Parameter values are the same as in Fig. 4 with the exception of the addition of demographic stochasticity. S’ = 0.23. $h^2 = 0.10$.

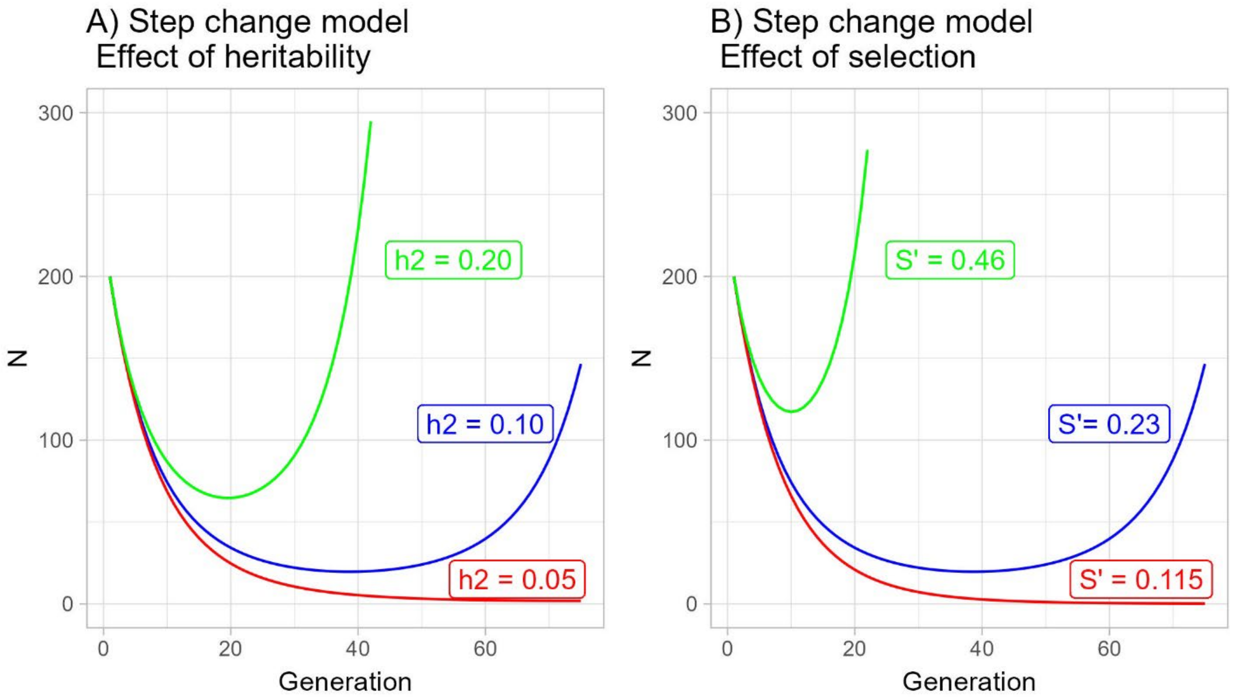

Supplementary Figure S2. Effects of heritability and selection on population dynamics in the *Step change model* with a shift to a constant extreme environment. A) Selection is kept at S’ = 0.23, and heritability varies. B) Heritability is kept at 0.10 and selection varies. Predicted population size is shown as a function of generation.
